# Interventions and Perinatal Outcomes Associated With Recipient Twin Cardiomyopathy in the Setting of Twin‐To‐Twin Transfusion Syndrome: A Systematic Review

**DOI:** 10.1002/pd.70141

**Published:** 2026-04-05

**Authors:** Briana Tolbert, Sandhya Chandrasekaran, Vanya Manthena, Gabrielle Sudilovsky, Andrew Rausch, Ryan E. Longman, Yves Ville, Ashish Premkumar

**Affiliations:** ^1^ Department of Obstetrics and Gynecology Biological Sciences Division The University of Chicago Chicago Illinois USA; ^2^ Biological Sciences Division Center for Interdisciplinary Inquiry and Innovation in Sexual and Reproductive Health The University of Chicago Chicago Illinois USA; ^3^ Biological Sciences Division Pritzker School of Medicine The University of Chicago Chicago Illinois USA; ^4^ Department of Obstetrics Fetal Medicine and Surgery Necker‐Enfants Malades Hospital APHP Paris France; ^5^ Department of Obstetrics, Fetal Medicine, and Surgery Paris Descartes University L'hôpital Necker‐Enfants malades (AP‐HP) Paris France

## Abstract

**Objective:**

To determine (1) Whether the presence and severity of recipient twin cardiomyopathy in the setting of twin‐to‐twin transfusion syndrome are associated with worse perinatal outcomes when compared to recipient twins without evidence of cardiomyopathy and (2) The optimal management strategy to reduce the likelihood of adverse perinatal outcomes when recipient twin cardiomyopathy is present.

**Methods:**

We included observational studies and randomized controlled trials of monochorionic‐diamniotic twin gestations affected by twin‐to‐twin transfusion syndrome, which reported the relationship between recipient twin cardiomyopathy and adverse perinatal outcomes. PubMed, CINAHAL, Scopus, Embase, and the Cochrane Central Register of Controlled Trials were queried from January 2004 to December 2025. The primary outcome was single and dual twin survival at 6 months of life. The secondary outcomes were the frequency of live birth of one or both twins; gestational age at the time of delivery; latency from diagnosis of twin‐to‐twin transfusion syndrome to delivery; birthweight of living twins; newborn length(s) of stay; need for neonatal intensive care unit admission; single and dual twin survival at 0 and 1 month of life; postnatal recipient twin cardiomyopathy (inclusive of right ventricular outflow tract obstruction); and frequency of neonatal complications at 6 months of life. Assessment of bias was performed using the Cochrane Risk‐of‐Bias and Newcastle‐Ottawa tools.

**Results:**

Of 207 abstracts available, 13 met the inclusion criteria. None of the studies reported the primary outcome defined by the systematic review, but did report on multiple secondary outcomes. Most manuscripts (10/13) used the Myocardial Performance Index to define recipient twin cardiomyopathy. One manuscript reported on the use of calcium channel blockers in addition to selective fetoscopic photocoagulation, and its relationship with adverse perinatal outcomes. Most studies exhibited a low risk of bias. Three out of 10 manuscripts using the Myocardial Performance Index demonstrated a relationship between the presence and severity of recipient twin cardiomyopathy and adverse perinatal outcomes. Two manuscripts demonstrated that the use of calcium channel blockers perioperatively may be associated with a reduced frequency of adverse perinatal outcomes.

**Conclusion:**

A small number of studies have assessed recipient twin cardiomyopathy's role in the risk of adverse perinatal outcomes or whether unique forms of treatment may improve the risk of perinatal outcomes. Further, studies suffer from a lack of long‐term neonatal follow‐up. Future studies should investigate whether other markers of recipient twin cardiac compromise are associated with adverse perinatal outcomes.

## Summary

- What is already known about this topic?
  ◦ Although the Quintero staging system is widely used to stage twin‐to‐twin transfusion syndrome, it is limited in its ability to assess the risk of adverse perinatal outcomes. Researchers have postulated whether the use of recipient twin cardiomyopathy (RTC) should be used to better prognosticate outcomes. Further, researchers have attempted to assess whether the treatment of RTC would improve perinatal outcomes.
- What does this study add?
  ◦ This study highlights the lack of literature surrounding the prognostic implications of the presence of RTC. Additionally, this study showcases the range of scoring systems used to assess RTC but also highlights the lack of research regarding which scoring system is the best to assess for RTC. Lastly, there is also a lack of literature comparing treatment modalities for RTC and their impact on improving perinatal outcomes.

## 1 Introduction

Monozygotic twins account for nearly 1 in 300 pregnancies, 2/3 of which are monochorionic diamniotic (MCDA). Up to 15% of MCDA twins will be affected by twin‐to‐twin transfusion syndrome (TTTS) due to placental vascular imbalances in arteriovenous anastomoses. The development of TTTS in pregnancy carries a poor prognosis as it is associated with a high frequency of perinatal morbidity and mortality if left untreated. [1].

The pathophysiology of TTTS is related to the abnormal vascular connections within the placenta, which leads to hypervolemia in the recipient twin and hypovolemia in the donor twin. [2, 3, 4] In addition to an imbalanced blood volume, TTTS can result in a series of compensatory cardiac responses in the recipient twin, which include increased preload, increased stretch on cardiac chambers, hypertension, cardiac hypertrophy and valvular regurgitation. [5, 6, 7, 8] The donor twin's hypovolemia also triggers an upregulation of the renin‐angiotensin system, which can exacerbate the recipient twin's hypertension and cardiomyopathy. [5].

Currently, the Quintero staging system is considered the gold standard for evaluating the severity of TTTS. [9] This staging system can be misleading, incorrectly suggesting that stages run on a continuum where I and II are less severe. [10, 11, 12] Michelfelder et al. found that up to 50% of recipient twins with stage I or II show signs of altered cardiac structure and function. [13] Given that the Quintero staging system does not account for nuanced evaluations of fetal cardiac dysfunction, and because it is unclear if the presence of recipient twin cardiomyopathy (RTC) is associated with a worse prognosis, the current gap in the classification system has made it challenging to determine whether the presence and/or severity of RTC is associated with worse perinatal outcomes.

The goal of our systematic review is twofold: (1) To investigate whether the presence and/or severity of RTC is associated with worse perinatal outcomes when compared to recipient twins without evidence of cardiomyopathy; and (2) To determine the optimal management strategy to reduce the likelihood of adverse perinatal outcomes when recipient twin cardiomyopathy is present.

## 2 Methods

The systematic review protocol was submitted to the International Prospective Register of Systematic Reviews (PROSPERO) on 23 September 2023 (ID#: CRD42023463097). We adhered to the Preferred Reporting Items for Systematic Reviews and Meta‐Analyses (PRISMA) 2020 guidelines for this manuscript. [14].

### 2.1 Eligibility Criteria

Observational cohort and randomized controlled studies which included individuals with monochorionic‐diamniotic twin gestations affected by RTC were eligible for inclusion if data related to the presence of cardiomyopathy and management strategies were disclosed.

### 2.2 Search Process

We searched PubMed, CINAHAL, Scopus, Embase, and the Cochrane Central Register of Controlled Trials for randomized controlled trials published in English from January 2004 to December 2025. The former date was chosen to coincide with the publication of the seminal Eurofetus trial, which set the standard for fetoscopic laser photocoagulation (FLP) as the standard of care for TTTS. [15] We also reviewed the bibliographies of all included studies to evaluate any additional eligible manuscripts. The search strategy is outlined in the Appendix.

### 2.3 Data Extraction

All titles and abstracts were imported into EndNote [16] in which duplicate entries were removed. All remaining titles and abstracts were uploaded to Rayyan [17] and assessed for inclusion or exclusion by three research team members (B.T., V.M., and S.C.). Any disagreements on whether to include an abstract were adjudicated by one member of the research team (A.P.). Two research team members (B.T. and S.C.) extracted all data using standardized forms. Two senior authors (Y.V., A.P.) adjudicated any issues with data abstraction and interpretation.

### 2.4 Assessment of Risk of Bias

Two members of the research team (B.T. and S.C.) independently reviewed each of the included studies using the Cochrane Collaboration risk‐of‐bias tool for RCTs and the Newcastle‐Ottawa risk‐of‐bias tool for cohort studies. [18, 19] A traffic‐light plot was generated using the robvis package in *R* (R Foundation for Statistical Computing, Vienna, Austria). [20].

### 2.5 Outcomes

The planned primary outcome for this study was single and dual twin survival at 6 months of life. The secondary outcomes examined the frequency of live birth of one or both twins; gestational age at the time of delivery; latency from diagnosis of TTTS to delivery; birthweight of living twins; newborn length(s) of stay; need for neonatal intensive care unit admission; single and dual twin survival at 0 and 1 month of life; postnatal RTC (inclusive of right ventricular outflow tract obstruction); and frequency of neonatal complications at 6 months of life. These outcomes were selected a priori as they adhere to consensus statements related to relevant perinatal outcomes for TTTS. [21] However, after data acquisition, none of the studies reported on neonatal outcomes beyond 1 month of life (i.e., “short‐term perinatal outcomes”). Therefore, we changed our primary outcome to short‐term perinatal outcomes.

### 2.6 Subgroup Analyses

Post‐hoc, we chose to review the planned primary outcome among individuals affected with Quintero stage IV TTTS, given the higher prevalence of cardiac dysfunction in the setting of hydrops fetalis.

### 2.7 Data Synthesis

Given the heterogeneity among the studies, we chose to synthesize the results in the form of a systematic review rather than a meta‐analysis.

## 3 Results

### 3.1 Study Selection and Characteristics

**FIGURE 1:**
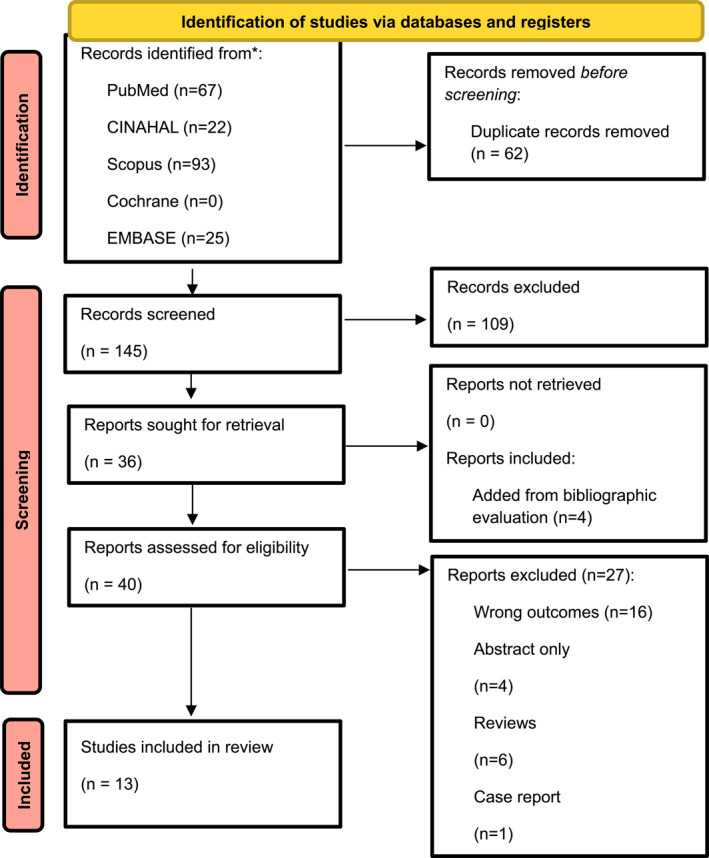
PRISMA 2020 flow diagram.

Of 207 abstracts for review, 40 met the inclusion criteria and were retrieved. Ultimately, 13 manuscripts were included in our study (Figure 1). [22, 23, 24, 25, 26, 27, 28, 29, 30, 31, 32, 33, 34] Characteristics and reported outcomes for all included studies are listed in Table 1. Supporting Information S1 summarizes all studies excluded from our analysis.

**TABLE 1:** Included studies.

Author (year)	Study design	Population (n)	Exposure	Primary outcome	Secondary outcome	How is RTC defined?	Results
Corroenne (2022) [1]	Retrospective cohort study	MCDA pregnancies complicated by TTTS undergoing FLP (*n* = 124)	Not applicable	Difference in echocardiographic findings between donor and recipient twin	Survival to birth of two, one, or no twins	Biventricular short and long axes; AV and outflow valve diameters; main pulmonary artery diameter, pulmonary artery peak systolic velocity	Fetal cardiac function was grossly abnormal in recipient twins when compared with donor twins (*p* < 0.01 for all cardiac findings). Fetal death following FLP occurred in 16% of recipient twins and 25% of donor twins, with dual twin death in 11% of cases. Intertwin differences in LV short axis dimension, aortic and mitral valve annulus diameter, and pulmonary artery peak systolic velocity associated with lower dual twin survival and survival of at least one twin.
Crombleholme (2010) [2]	Case control study	MCDA pregnancies complicated by TTTS undergoing FLP, matched by gestational age and Cincinnati stage (*n* = 141)	FLP with nifedipine (10–20 mg every 6 h), beginning 24–48 h prior to FLP and continuing until postoperative resolution of RTC or delivery	Recipient and donor survival to birth	Fetal survival to postoperative fetal echocardiogram (3–5 days after FLP), pre‐ and post‐operative changes in MPIs, maternal tolerance to nifedipine, maternal demographics, and composite maternal morbidity (i.e., death, spinal headache, bleeding requiring transfusion, trocar site infection, chorioamnionitis, pulmonary edema, or pulmonary embolism)	Defined by AVVR, degree of right and left ventricular hypertrophy, and MPI	There was a significant increase in overall fetal survival to birth between nifedipine‐treated cases and control subjects (237/284 [83%] vs. 232/308 [75%]; *p* = 0.015). There was no difference in the survival of 1 or both twins. Fetal recipient twin survival to the postoperative fetal echocardiographic assessment was significantly higher in the perioperative nifedipine group. There was a significant increase in recipient twin survival in stages IIIA‐B among those treated with perioperative nifedipine.
Delabaere (2016) [3]	Retrospective cohort study	MCDA pregnancies complicated by TTTS undergoing FLP (*n* = 106)	Postnatal survival at 30 days of life	Donor and recipient twin measurements of cardiac functioning up to 48 h pre‐operatively and 28 days post‐operatively	Not applicable	Shortening fraction, MPI, cardiomegaly, CHOP score, cardiac hypertrophy	30‐day survival was associated with pre‐operative recipient RV‐MPI, cardiac hypertrophy, and CHOP score. Recipient 30‐day survival rate was associated with post‐operative RV and LV MPI and CHOP score. No significant association between donor survival and hemodynamic preoperative measurements. 30‐day survival was associated with post‐operative donor twin RV MPI.
Delabaere (2018) [4]	Retrospective cohort study	MCDA pregnancies complicated by TTTS undergoing FLP (*n* = 112)	IUFD of one or both twins	IUFD of one or both twins 24 h and 7 days after FLP	Not applicable	MPI in both ventricles, cardiomegaly, recipient twin myocardial hypertrophy, and CHOP score	Frequency of IUFD at 24 h after FLP were 15.3% and 2.7% for 1 and 2 fetuses, respectively, representing 59% of all cases of spontaneous IUFD. IUFD within 7 days occurred in 18.9% and 3.6% of cases, representing 74.4% of all cases of spontaneous IUFD. Donor twin IUFD at 24 h and 7 days after FLP was not associated with cardiac ultrasonographic findings; however, Increased recipient RV‐MPI z‐score was associated with recipient twin IUFD at 24 h and 7 days after FLP.
Eixarch (2013) [5]	Retrospective cohort study	MCDA pregnancies complicated by TTTS undergoing FLP at 3 sites (*n* = 215)	IUFD of either donor or recipient twin within 1 week of FLP	Not applicable	Not applicable	MPI	IUFD within the first week of surgery occurred in 7.9% (17/215) of recipients and in 15.3% (33/215) of donors. There were no significant differences in MPI values between surviving and non‐surviving twins.
Finneran (2017) [6]	Retrospective cohort study	MCDA pregnancies complicated by TTTS undergoing FLP with evidence of preoperative RTC (*n* = 43)	Not applicable	Change in pre‐and post‐operative MPI for both left and right ventricles.	Survival at 24 h and 7 days after FLP, as well as at birth	MPI	There was a difference of −0.15 ± 0.12 and a 24% reduction in the LV MPI when comparing pre‐operative MPI to post‐operative MPI. There was a difference of −0.16 and a 24% reduction in the RV MPI when comparing pre‐operative MPI to post‐operative MPI. Of the 39 patients with both preoperative and postoperative MPI data available, 37 (95%) showed improvement of cardiac function in at least one ventricle, and 22 (56%) showed normal MPI measurements in both the RV and LV. There was no significant association between MPI and donor or recipient survival.
Gapp‐Born (2014) [7]	Retrospective cohort study	MCDA pregnancies complicated by TTTS undergoing FLP (*n* = 105)	CHOP and MPI scores	Recipient twin demise	Change in pre‐and post‐operative MPI; correlation between Quintero staging, MPI, and CHOP score	CHOP score and MPI	The risk of recipient twin demise was significantly higher when the MPI z‐score was > 1.645 (34.5% vs. 10.6, *p* = 0.004) and when the CHOP cardiovascular score was ≥ 3 (39.5% vs. 12.9%, *p* = 0.002); the former was no longer significant after adjusting for relevant confounding variables. There was a positive correlation between Quintero stage and CHOP score (rho correlation coefficient 0.56. *p* < 0.001) and MPI (rho correlation coefficient 0.33, *p* = 0.01).
Habli (2008) [8]	Retrospective cohort study	MCDA pregnancies complicated by TTTS undergoing FLP (*n* = 75)	FLP	Improved MPI, defined as greater than 10% interval decrease in either left and/or right MPI; normalization of venous and/or arterial Doppler findings; presence/absence of mild or greater AVVR; and recipient twin survival at time of birth	Not applicable	MPI	An improvement of LV MPI after FLP is associated with a significant increase in recipient twin survival to birth, when compared with recipient twins with no improvement after FLP (100 *v*. 86.1%, *p* < 0.01). There was no significant difference in recipient survival between those with or without interval improvements in RV MPI.
Maskatia (2026) [9]	Retrospective cohort study	MCDA pregnancies complicated by stage III – IV TTTS undergoing FLP (*n* = 285)	FLP	Donor twin demise, recipient twin demise (within 30 days after delivery)	Effects of confounders on recipient and donor demise (fetal weight, gestational age at time of echocardiogram, fetal sex, use of perioperative nifedipine)	Cardiac output, MPI, ventricular systolic dysfunction and valvular regurgitation	On regression modeling, donor twin demise was reduced with the use of preoperative nifedipine. Further, recipient combined cardiac output was associated with an increased risk of donor demise. Recipient demise was reduced with the use of perioperative nifedpine. Recipient combined cardiac output and LVI were significantly associated with recipient twin demise.
Skupski (2010) [10]	Retrospective cohort study	MCDA pregnancies complicated by TTTS undergoing FLP at 8 centers (*n* = 466)	Donor and recipient death, either in utero or neonatally	Preoperative donor and recipient twin measurements of cardiac functioning	Not applicable	Global cardiac dysfunction (abnormal MPI, ventricular dyskinesia, abnormal LV or RV ejection fraction, abnormal cardiac scoring)	On bivariate analysis, global recipient cardiac dysfunction was associated with increased risk of recipient twin fetal demise (35 v. 40%), but not neonatal demise. There was no association between global cardiac dysfunction and either donor death in utero or neonatally. On regression modeling, donor or recipient twin global cardiac dysfunction was not significantly associated with fetal or neonatal death.
Stirnemann (2010a) [11]	Prospective cohort study	MCDA pregnancies complicated by TTTS undergoing FLP (*n* = 215)	CHOP stage	Neonatal survival of neither, one, or both twins	Not applicable	CHOP score	Overall survival was 33% and 55% for one and both twins, respectively, yielding a perinatal survival rate of at least one twin of 88%. Rates of dual survival by CHOP stages 1–3 were 54%, 64%, and 83%; survival of one twin was 72%, 86%, and 83%. However, the relationship between CHOP stage and survival was not significant (*p* = 0.62).
Stirnemann (2010b) [12]	Prospective cohort study	MCDA pregnancies complicated by TTTS undergoing FLP (*n* = 107)	Not applicable	Survival up to 28 days after delivery	Not applicable	Cardiothoracic ratio, shortening fractions, wall thickness, combined cardiac output, cardiac index, MPI, AVVR	Overall, 85% had at least one twin surviving and both twins survived in 51% of pregnancies. Perinatal survival rate was 63% in donor and 73% in recipient twins. Using a combination of shortening fraction, MPI, and cardiac index, three profiles were created, corresponding to 39.7 (near‐normal cardiac functioning), 49.2 (diastolic and global myocardial dysfunction), and 11.1% (systolic, diastolic, and global myocardial dysfunction) of recipient twins. However, perinatal survival of one or both twins was not associated with any of the cardiac profiles.
Shah (2008) [13]	Retrospective cohort study	MCDA pregnancies complicated by TTTS, undergoing either FLP (*n* = 30) or amnioreduction (*n* = 32)	Pre‐procedure CVPS	Recipient twin survival to 30 days of life	Not applicable	CVPS	Overall neonatal survival was 61%; recipient twin survival was 58%. Grouped by CVPS, recipient twin survival was the highest for those with CVPS of 9 (50%) and 10 (74%). Among the components of the CVPS, AVVR was associated a lower likelihood of the primary outcome (39 *v*. 69%, *p* = 0.02).

### 3.2 Risk of Bias

**FIGURE 2:**
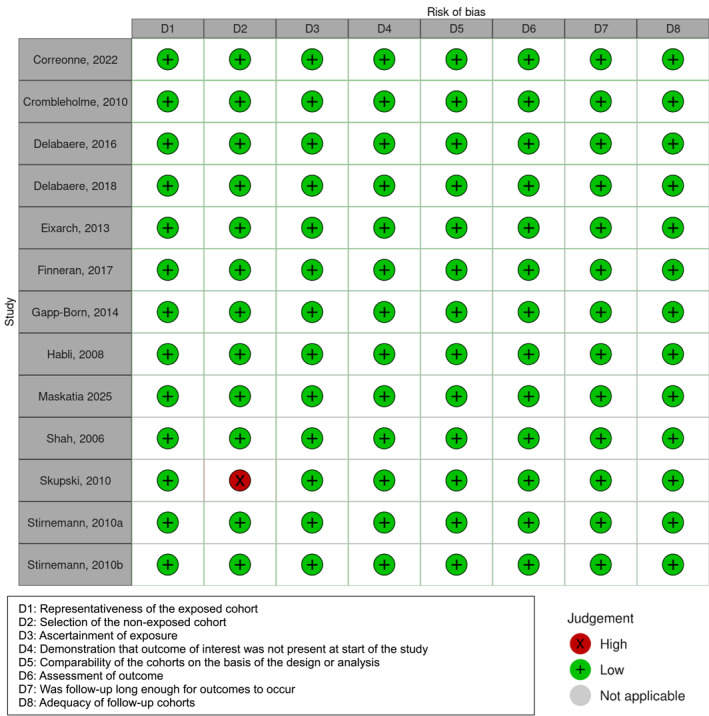
Risk‐of‐bias traffic light plot.

Figure 2 depicts a traffic‐light pilot of the risk of bias of all included studies. Overall, most studies included in the review illustrated a low risk of bias. Only one study had an elevated risk of bias in the selection of the non‐exposed cohort (Figure 2).

### 3.3 Synthesis of Results

#### 3.3.1 RTC and Adverse Perinatal Outcomes

**FIGURE 3:**
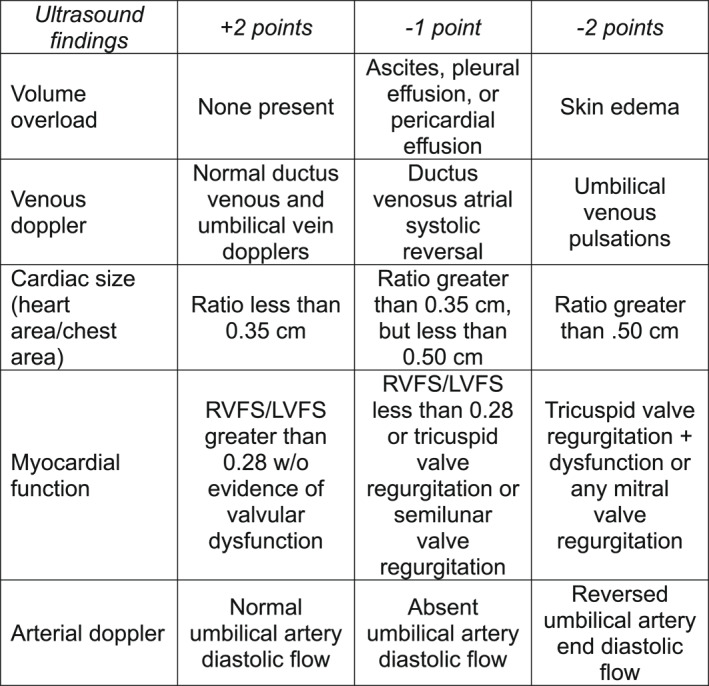
Cardiovascular profile score.

**FIGURE 4:**
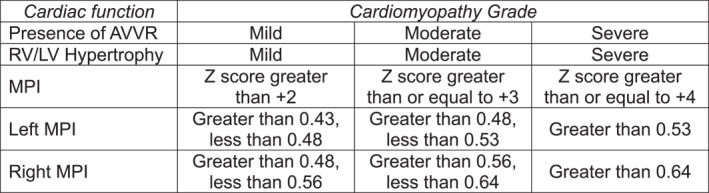
Cincinnati Staging system.

**FIGURE 5:**
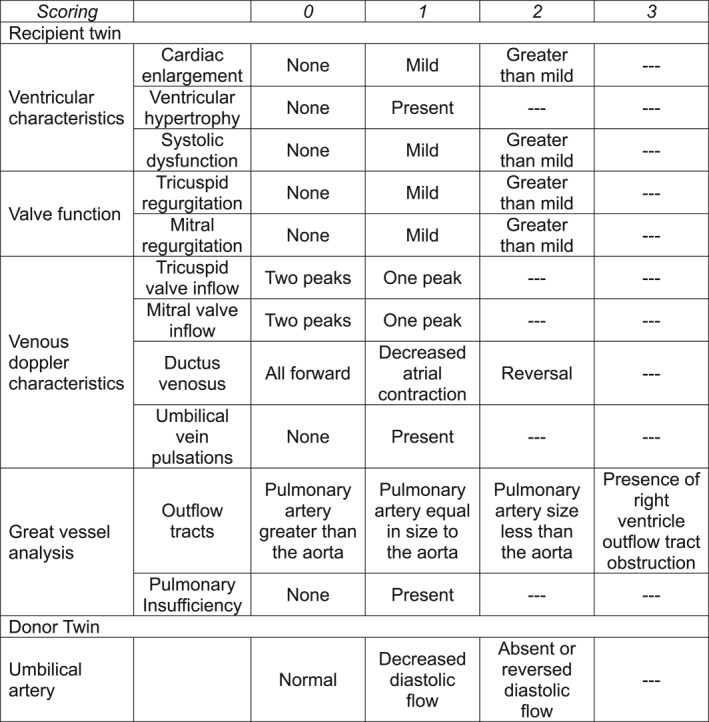
CHOP cardiovascular score.

**FIGURE 6:**
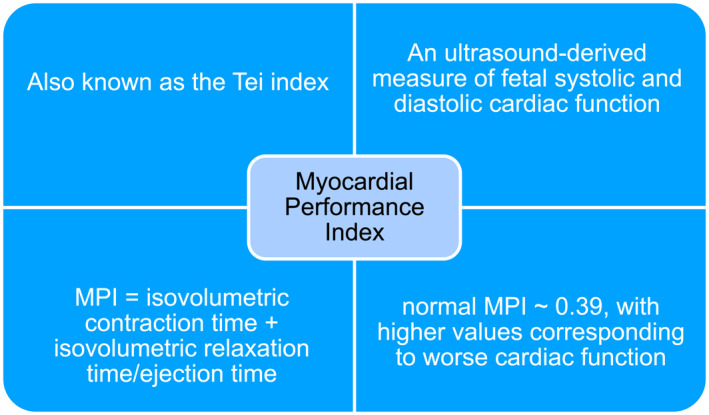
Myocardial performance index.

Of the 13 manuscripts, 10 used the MPI scoring system to define RTC, while 4 studies used the CHOP scoring system, and one study used the CVPS scoring system to define RTC (Figures 3, 4, 5, 6).

Five out of 10 manuscripts reporting MPI suggest that a higher MPI score is associated with an increased risk of recipient twin demise when compared with a lower MPI score, resulting in less cardiac dysfunction. Gapp‐Borne and colleagues noted that in MCDA twins complicated by TTTS and treated with FLP, higher MPI scores were correlated with a higher risk of recipient twin fetal demise. [28] Delabaere and colleagues noted that in MCDA twins complicated by TTTS, the risk of fetal demise for the recipient twin at 24 h and 7 days after FLP was higher in those with higher MPI score. [25] In another paper, Delabaere and colleagues also noted that in MCDA twins complicated by TTTS, when comparing pre‐operative and post‐operative MPI score, a low pre‐operative MPI was associated with high risk of fetal survival in the recipient twin 30 days after FLP. [24] No significant relationship was found between recipient twin survival and postoperative cardiac measurements. Habli and colleagues noted that change in left ventricular MPI after FLP was associated with a significant increase in recipient twin survival to birth, when compared with recipient twins with no improvement after FLP (100 *v*. 86.1%, *p* < 0.01). [29].

Only one study used the CVPS as a risk‐stratifying tool to assess its relationship with fetal outcomes. Shah and colleagues showed that low CVPS correlated with a higher risk of fetal neonatal death in the recipient twins. [33] No study reporting the CHOP scoring system linked the presence of RTC with adverse perinatal outcomes.

#### 3.3.2 Interventions for the Management of RTC and Adverse Perinatal Outcomes

One study focused on comparing management strategies and their association with fetal and neonatal outcomes. As many of the studies focused on RTC standardly use FLP as a form of treatment, no manuscript in our systematic review evaluated the role of expectant management in the setting of RTC, given the high concordance between RTC and higher Quintero stages of TTTS. In an observational study, Crombleholme and colleagues showed that the administration of CCBs (20 mg of nifedipine every 6 h for 24–48 h before undergoing FLP and continuation until resolution of RTC or delivery) in addition to FLP, improved recipient twin survival to birth when compared to FLP alone. In a post‐hoc analysis, the authors demonstrated a survival advantage for stage IIIB recipients who were exposed to nifedipine (93% *v*. Seventy‐five percent, *p* = 0.039), and not stage IIIA or stage IIIC and IV recipient twins.

In a retrospective cohort study of pregnancies undergoing FLP for stage III‐IV TTTS, Maskatia and colleagues demonstrated that preoperative exposure to nifedipine was associated with a reduction in the frequency of donor twin demise after considering other cardiac parameters, such as recipient twin combined cardiac output. Furthermore, recipient twin demise was significantly less likely with the use of perioperative nifedipine when considering recipient twin combined cardiac output and LVI. [34].

#### 3.3.3 Post‐Hoc Analysis of RTC and Adverse Perinatal Outcomes Among Stage IV TTTS

One out of 13 studies (7.6%) directly reported the relationship between RTC and adverse perinatal outcomes concerning stage IV TTTS. Gapp‐Born et al. showed that of 4 fetuses with Quintero stage IV TTTS, 100% had an MPI z‐score > 1.645 and a CHOP score greater than or equal to 3. Importantly, there was a 75% frequency of recipient twin loss up to 7 days postnatally. [28].

## 4 Discussion

### 4.1 Principal Findings

In this systematic review, we illustrate inconclusive findings related to the role of RTC in TTTS, both as a harbinger of adverse perinatal outcomes and as a target for potential interventions. We believe that this is due to inconsistent definitions of RTC and the variable duration of neonatal follow‐up.

### 4.2 Comparison With Existing Literature

Systematic review and meta‐analytic data suggest that Quintero staging, the most commonly‐employed technique for ascertaining the severity of TTTS, is poorly associated with the prediction of adverse perinatal outcomes. [12] Therefore, researchers have sought to assess the role of other ultrasonographic markers, such as RTC, to risk‐stratify those pregnancies at the highest chance of adverse perinatal outcomes. Our data show that while certain data may point toward the role of RTC in predicting adverse perinatal outcomes, the paucity of standardization of both the exposure and outcomes between studies makes any conclusion difficult to derive. These findings call into question the recent Delphi consensus on TTTS from the North American Fetal Therapy Network, which advocates using RTC (i.e., tricuspid regurgitation) as part of TTTS staging. [35].

The optimal method of defining RTC is difficult to ascertain in the literature as the reported studies use a variety of methods to define the presence and/or severity of RTC. Importantly, Stirnemann and colleagues showed that abnormal peripheral Doppler evaluation of the recipient and donor twins (e.g., pulsatile umbilical vein, abnormal umbilical artery Doppler waveforms) were the main contributors to CHOP scoring among pregnancies diagnosed with higher Quintero stages. [32] Although this finding is intuitive, since peripheral Doppler waveform abnormalities are the hallmark of higher Quintero stages, the authors also illustrate a lack of correlation between CHOP score and perinatal outcomes. Data from Maskatia and colleagues demonstrate that recipient twin combined cardiac output and LVI may play a role in predicting donor and recipient demise, respectively, in stage III‐IV TTTS. [34] However, based on the extant data, it is unknown which, if any, markers of RTC may enhance the prognostic capabilities afforded by Quintero staging alone, both in terms of perinatal survival and long‐term health outcomes.

Furthermore, the role of RTC in the setting of Quintero stage I disease is difficult to interpret. Meta‐analytic data do not demonstrate improvement in perinatal outcomes when FLP is employed for treatment. [36] Limited data are available to assess the presence of RTC in the setting of Quintero stage I TTTS and its relation to adverse pregnancy outcomes. [13, 32, 37, 38] However, the definition of RTC and measured outcomes differ between studies. Further research into the prevalence and severity of RTC in early‐stage TTTS and its relationship with adverse perinatal outcomes is warranted.

Although two studies in our systematic review evaluated perioperative medical management in the setting of RTC [23, 34], other non‐procedural interventions have historically been documented in case reports. Unsurprisingly, conclusive evidence to drive clinical utility is lacking. Maternal digoxin therapy has been published in early reports, which was used in conjunction with procedural interventions with positive outcomes. [39] However, given the lack of evidence and unclear mechanism of action in addressing the specifics of fetal cardiac dysfunction related to TTTS, this medication is no longer used. One study in our review evaluating adjunctive medication therapy, nifedipine, demonstrated promising efficacy in improving perinatal outcomes. [23] This finding was further demonstrated in the predictive modeling conducted by Maskatia and colleagues. [34] Further work, ideally conducted in the form of a prospective randomized trial with an increased sample size and standardized outcome assessment, will be needed to underscore these initial findings.

### 4.3 Strengths and Limitations

There are multiple strengths to our systematic review. We evaluated multiple databases with a variety of search terms for eligible studies. Second, we limited our search to trials conducted since 2004 after the seminal Eurofetus randomized controlled trial, to account for the standardization of selective fetoscopic laser photocoagulation as the mainstay of treatment for TTTS. [15] Furthermore, while other systematic reviews have focused on the presence of right heart outflow tract obstruction or the relationship between ultrasonographic markers and post‐FLP demise, no systematic reviews to date have focused on the role of RTC and adverse perinatal health outcomes or the optimal management strategy for RTC. [40].

A limitation encountered during this systematic review was the lack of unifying scoring systems for RTC across the included studies. The original purpose of the Quintero staging system was to provide non‐invasive parameters to gauge the burden of TTTS. [9] The CHOP scoring system, which uses cardiovascular parameters to tailor staging, was created to better characterize disease severity and magnitude of impact on neonatal outcomes. [41] However, intriguingly, even this streamlined metric illustrates discrepant results regarding its prognostic utility on neonatal outcomes, including survival.

Despite our a priori designated primary outcome being single and dual twin survival at 6 months of life, none of the papers included in our review followed neonates over that length of time. The most common time points referenced in these studies were 24–48 h post‐laser treatment, 1 week following delivery, and 4 weeks following delivery. However, given documented evidence of fetal cardiovascular remodeling persisting up to 6 months in infants, longitudinal follow‐up around this time frame is crucial in truly understanding the cardiovascular pathophysiological sequelae of TTTS in a meaningful manner. Adhering to Delphi consensus guidelines in future studies will be critical to assess the long‐term health outcomes related to RTC. [21].

## 5 Conclusion

In this systematic review, we found inconclusive evidence as to the relationship between RTC, and the role of potential interventions aimed at ameliorating RTC, and adverse perinatal outcomes. Future studies should be conducted, ideally in a prospective fashion with rigorous, peer‐reviewed, and reproducible definitions of the exposure and relevant outcomes, to assess the relationship between RTC and adverse perinatal outcomes.

## Funding

The authors have nothing to report.

## Ethics Statement

The authors have nothing to report.

## Consent

The authors have nothing to report.

## Conflicts of Interest

The authors declare no conflicts of interest.

## Supporting information


Supporting Information S1

## Data Availability

The data that support the findings of this study are available from the corresponding author upon reasonable request.
