# Supplementary material for: Interventions and Perinatal Outcomes Associated With Recipient Twin Cardiomyopathy in the Setting of Twin‐To‐Twin Transfusion Syndrome: A Systematic Review
Source: Prenat Diagn. 2026 Apr 5;46(8):1159–72. doi: 10.1002/pd.70141 (PMC13377285; doi:10.1002/pd.70141)
Supplement: Supplementary file 1 — Supporting Information S1 [file PD-46-1159-s001.docx]

APPENDIX

Search strategy

Search 9/11-15/23 (repeated 1/6/26)

PubMed (n=67)

(“Cardiomyopathies"[Mesh] OR “Fetal Heart”[Mesh] OR “Ultrasonography, Prenatal” [Mesh]) AND (“Fetofetal Transfusion” [Mesh] OR “Disease Progression” [Mesh] OR “Severity of Illness Index” [Mesh]) AND (“Fetoscopy” [Mesh] OR “Laser Coagulation” [Mesh] OR “Nifedipine” [Mesh] OR “Vasodilator Agents”) AND "Adult"[Mesh] AND "Female"[Mesh] AND “Humans” [Mesh] AND “Pregnancy” [Mesh]

CINAHAL (n=22)

(MM “Cardiomyopathies” OR MM “Fetal heart” OR “Ultrasonography, Prenatal”) AND (MM “Fetofetal Transfusion” OR MM “Disease Progression” OR MM “Severity of Illness Indices”) AND (MM “Fetoscopy” OR “MM “Laser Coagulation” OR MM “Nifedipine” OR MM “Vasodilator Agents”) AND (MM “Pregnancy”)

SCOPUS (n=93)

INDEXTERMS((“Cardiomyopathies" OR “Fetal Heart” OR “Ultrasonography, Prenatal”) AND (“Fetofetal Transfusion” OR “Disease Progression” OR “Severity of Illness Index”) AND (“Fetoscopy” OR “Laser Coagulation” OR “Nifedipine” OR “Vasodilator Agents”) AND "Adult" AND "Female" AND “Humans” AND “Pregnancy”) AND ( LIMIT-TO ( DOCTYPE,"ar" ) OR LIMIT-TO ( DOCTYPE,"cp" ) ) AND ( LIMIT-TO ( SUBJAREA,"MEDI" ) )

Cochrane Trials (n=0)

ID Search Hits

#1 MeSH descriptor: [Fetal Heart] explode all trees 140

#2 MeSH descriptor: [Cardiomyopathies] explode all trees 2480

#3 MeSH descriptor: [Ultrasonography, Prenatal] explode all trees 699

#4 MeSH descriptor: [Fetofetal Transfusion] explode all trees 31

#5 MeSH descriptor: [Disease Progression] explode all trees 9455

#6 MeSH descriptor: [Severity of Illness Index] explode all trees 24278

#7 MeSH descriptor: [Fetoscopy] explode all trees 38

#8 MeSH descriptor: [Laser Coagulation] explode all trees 765

#9 MeSH descriptor: [Nifedipine] explode all trees 2241

#10 MeSH descriptor: [Vasodilator Agents] explode all trees 4212

#11 MeSH descriptor: [Adult] explode all trees 586627

#12 MeSH descriptor: [Female] explode all trees 582353

#13 MeSH descriptor: [Humanism] explode all trees 21

#14 MeSH descriptor: [Pregnancy] explode all trees 31435

#15 (#1 OR #2 OR #3 OR #4 OR #5 OR #6) AND (#7 OR #8 OR #9 OR #10) AND #11 AND #12 AND #13 AND #14 0

EMBASE (n=25)

('fetoscopy'/exp OR 'vasodilator'/exp OR 'laser photocoagulation'/exp OR 'nifedipine'/exp OR 'laser coagulation'/exp) AND ('cardiomyopathy'/exp OR 'fetal heart'/exp) AND 'twin twin transfusion syndrome'/exp AND 'pregnancy'/exp

Total sample = 207

Duplicates removed = 62

Total sample for review n=145

Supplementary Table 1. Excluded manuscripts

| Author (year) | Study Design | Population (n) | Exposure | Primary Outcome | Secondary Outcome | How is RTC defined? | Reason for exclusion |
| --- | --- | --- | --- | --- | --- | --- | --- |
| Barrera (2004) | Prospective Cohort Study | MCDA pregnancies complicated by TTTS undergoing amnioreduction (n=54) | Amnioreduction | ﻿Recipient twin right and left ventricular wall thickness, diameters, systolic and diastolic function, valve regurgitation, and structural cardiac defects | ﻿Not applicable | Not defined | Excluded from study due to RTC not being defined and due to results not being applicable to primary or secondary outcomes for our study |
| Barrera (2006) | Prospective Cohort Study | MCDA pregnancies complicated by TTTS undergoing FLP (n=22) | SFLP | Systolic & diastolic function of recipient twin measured with fetal ECHO | Not applicable | Not defined | Excluded from study due to RTC not being defined and due to results not being applicable to primary or secondary outcomes for our study |
| Chaszczewski (2025) | Retrospective Cohort Study | MCDA twins complicated by TTTS undergoing FLP (n=198) | FLP | UA PI, MCA PI, CPR and CHOP TTTS CV Score elements | Not applicable | CHOP scoring | Excluded from study due results not being applicable to primary or secondary outcomes for our study |
| Diehl (2014) | review | Not applicable | none | n/a (review) | n/a | Not defined | n/a |
| Dunn (2016) | Prospective Cohort Study | MCDA pregnancies complicated by TTTS (n=19) | SFLP | ﻿The metabolomics profile of the amniotic fluid from the recipient sac of TTTS pregnancies was assessed using ultra high-performance liquid chromatography- mass spectrometry. | Not applicable | Left ventricle and right ventricle myocardial performance index (MPI). | Excluded from study due to results not being applicable to primary or secondary outcomes for our study |
| Eschbach (2018) | Prospective Cohort Study | MCDA pregnancies complicated by TTTS (n=124) | RVOTO (right ventricle outflow tract obstruction) | Prenatal/postnatal RVOTO | Not applicable | RVOTO | Excluded due to absence of data linking presence/absence of RVOTO to neonatal outcomes |
| Habli (2009) | Review | Not applicable | none | n/a (review) | n/a | Not defined | n/a |
| Habli (2023) | Abstract only - Retrospective study | TTTS cases (75 -Stage IV) | SFLP | prenatal cardiac predictors of survival in TTTS | not defined (abstract only) | MPI | Cardiomyopathy was seen in  93% (70/75) of the hydropic recipient twins. Overall fetal survival at birth was 86% (129/150), while  97% (73/75) of pregnancies had at least one survival. Survival at  birth was 84% (63/75) for recipient and 88% (66/75) for donor.  Recipient non-survivors compared to recipient survivors had a  significantly higher cardiothoracic ratio; higher incidences of moderate to severe left ventricular  systolic dysfunction (55% vs. 15%, P=0.007), as well as functional  pulmonary atresia (64% vs. 18%, p= 0.003). There were no significant differences regarding other cardiac parameters between  the two groups. |
| Hecher (2018) | Review | Not applicable | none | n/a (review) | n/a | Not defined | n/a |
| Herberg (2005) | Prospective Cohort Study | MCDA pregnancies complicated by TTTS (73) | SFLP | Cardiac function in twins | Congenital heart disease | Not defined | Excluded from study due to RTC not being defined and due to results not being applicable to primary or secondary outcomes for our study |
| Khalek (2013) | Review | Not applicable | None | n/a (review) | n/a | Not defined | n/a |
| Lewi (2008) | Prospective Cohort Study | MCDA pregnancies complicated by TTTS (221) | None | Not applicable (observation study) | Not applicable | Not defined | Excluded due to findings not being applicable to primary or secondary outcomes for our study |
| Michelfelder (2007) | Cross-sectional Retrospective Study | MCDA pregnancies complicated by TTTS (n=42) | fetal cardiac assessment | Cardiac structural & functional changes | Not applicable | Various parameters of cardiac function were used to define cardiomyopathy as well as the myocardial performance index (MPI) | Excluded from study due to RTC not being defined and not being applicable to primary or secondary outcomes for our study |
| Moussa (2023) | Abstract only - Retrospective study | TTTS cases (30) | SFLP | ASQ scores (scoring developmental domains, including communication,  gross motor, fine motor, personal-social, and problem solving). Scoring completed by Caregiver | not defined (abstract only) | not stated in abstract | despite earlier gestational age at delivery (32.6 vs 34.0 weeks), TTTS patients  s/p SFLP and treated with nifedipine had demonstrated higher ASQ  scores in the categories of communication, gross motor, fine motor,  and problem solving |
| Ortiz (2015) | Prospective Cohort Study | MCDA pregnancies complicated by TTTS (n=24) | SFLP | MAPSE/TAPSE | Not applicable | Not defined | Excluded from study due to RTC not being defined |
| Papanna (2011) | Prospective Cohort Study | MCDA pregnancies complicated by TTTS (n=20) | SFLP | Serial TEI index changes pre-operative, intra-operative, and post-operative (12h, 24h) of the recipient fetus | Not applicable | Not defined | Excluded from study due to RTC not being defined in addition to results not being applicable to primary or secondary outcomes for our study |
| Pruetz (2009) | Case Report | MCDA pregnancies complicated by TTTS (n=1) | SFLP | circular shunt/RV dysfunction in the recipient twin | Not applicable | Not defined | Excluded due to study design |
| Pruetz (2011) | Prospective Cohort Study | MCDA pregnancies complicated by TTTS (n=50) | SFLP | prevalence of postnatal heart disease among both donor and recipient survivors following laser therapy for TTTS | Not applicable | Not defined | Excluded from study due to RTC not being defined in addition to results not being applicable to primary or secondary outcomes for our study |
| Tabbah (2023) | Abstract only - Prospective study | TTTS cases (56) | SFLP | correlation of amniotic fluid levels of vasopressin with severity of recipient cardiomyopathy in TTTS | not defined (abstract only) | Cincinnati TTTS staging system | 9 recipient amniotic fluid samples were analyzed and com-  pared to 7 controls. Distribution of stages was stage III (n=1), IIIa  (n=7), IIIb (n=12), IIIc (n=20), and IV (n=9). As compared to controls, there was no significant difference in  recipient AVP levels among groups (Figure). No trend in AVP con-  centrations by stage was identified. |
| Takano (2019) | Prospective Cohort Study | MCDA pregnancies complicated by TTTS (n=56) | SFLP | Impact on diastolic function | Not applicable | Not defined | Excluded from study due to RTC not being defined in addition to results not being applicable to primary or secondary outcomes for our study |
| Takano (2022) | Prospective Cohort Study | MCDA pregnancies complicated by TTTS (n=50) | Fetal cardiac dysfunction | Correlation of afNT-proBNP levels with UA, DV, and MCA PI | Not applicable | Increased cardiac load | Excluded from study due to results not being applicable to primary or secondary outcomes for our study |
| Ting (2024) | Retrospective Cohort Study | Live donor and recipient neonates complicated by TTTS who underwent FLP (n=11) | Donor or recipient status | Echocardiographic data at 2 weeks of life | Not applicable | Not discussed | Excluded from the study due to inability to capture pre-FLP evidence of RTC |
| Van Mieghem (2009) | Prospective Cohort Study | MCDA pregnancies complicated by TTTS (n=23) | SFLP | Cardiac function (LV/RV MPI, AV flow pattern, DV a-wave, UV pulsations) | Not applicable | Not defined | Excluded from study due to RTC not being defined in addition to results not being applicable to primary or secondary outcomes for our study |
| Van Mieghem (2010) - B | Prospective Case Control | MCDA pregnancies complicated by TTTS (n=17) | None | ﻿Peak systolic strain, strain rate, velocity, and displacement were calculated, corrected for gestational age | Not applicable | ﻿Peak systolic strain, strain rate, velocity, and displacement | Excluded due to results not being applicable to primary or secondary outcomes for our study |
| Van Mieghem (2010) - B | Review | Not applicable | None | n/a (review) | n/a | Not defined | n/a |
| Wellen (2018) - A | Retrospective Cohort Study | MCDA pregnancies complicated by TTTS (n=76) | SFLP | Pulmonary artery abnormalities and right ventricle outflow tract obstruction in recipient TTTS twins | Not applicable | Grouped by extent of RVOTO | Excluded due to results not being applicable to primary or secondary outcomes for our study |
| Wellen (2018) - B | Abstract only - Retrospective study | TTTS cases (76) | SFLP | impact of LP on PA abnormalities and RVOTO in recipient TTTS twins | not defined (abstract only) | CHOP score | Of 76 twin pairs with TTTS who underwent LP at our center, 28 (36%) had right-sided outflow  abnormality. Prior to LP, 15 had a PA equal in size to the aorta (group 1), 5 had a PA smaller than the  aorta (group 2) and 8 had frank RVOTO (group 3). All group 1 and group 2 fetuses had complete resolution by 4 wks  after LP. In group 3, 1 patient with “functional” pulmonary atresia (no antegrade flow but with pulmonary  insufficiency) had complete resolution 1 day after LP. Five patients (63%) with RVOTO at diagnosis did  not respond to LP, of whom four required a catheter-based intervention after birth. All four remained  biventricular repairs. There were 11 cases of fetal demise (6 donor, 5 recipient). |
| Wilner (2021) | Prospective Cohort Study | MCDA pregnancies complicated by TTTS (n=14) | expression of mRNAs in amniotic fluid | differential expression of mRNA between twin with cardiomyopathy and those without | Gestational age at delivery, latency from procedure to delivery, neonatal survival | Right ventricular myocardial performance index of the recipient fetus of > 4 z- scores | Excluded due to results not being applicable to primary or secondary outcomes for our study |
| Zaretsky (2014) | Review | Not applicable | none | n/a (review) | n/a | Not defined | n/a |
